# Supplementary material for: Label-free single-cell RNA multiplexing leveraging genetic variability
Source: Nat Commun. 2024 Dec 5;15:10612. doi: 10.1038/s41467-024-54270-6 (PMC11621319; doi:10.1038/s41467-024-54270-6)
Supplement: Supplementary file 1 — Supplementary Information [file 41467_2024_54270_MOESM1_ESM.pdf]

Supplementary Information

Label-free single-cell RNA Multiplexing leveraging Genetic Variability

Konrad Hoeft, Tore Bleckwehl, David Schumacher, Hyojin Kim, Robert Meyer, Qingqing Long, Ling Zhang, Christian Möller, Marian C Clahsen-van Groningen, Anne Babler, Turgay Saritas, Ingo Kurth, Hendrik Milting, Sikander Hayat, Rafael Kramann

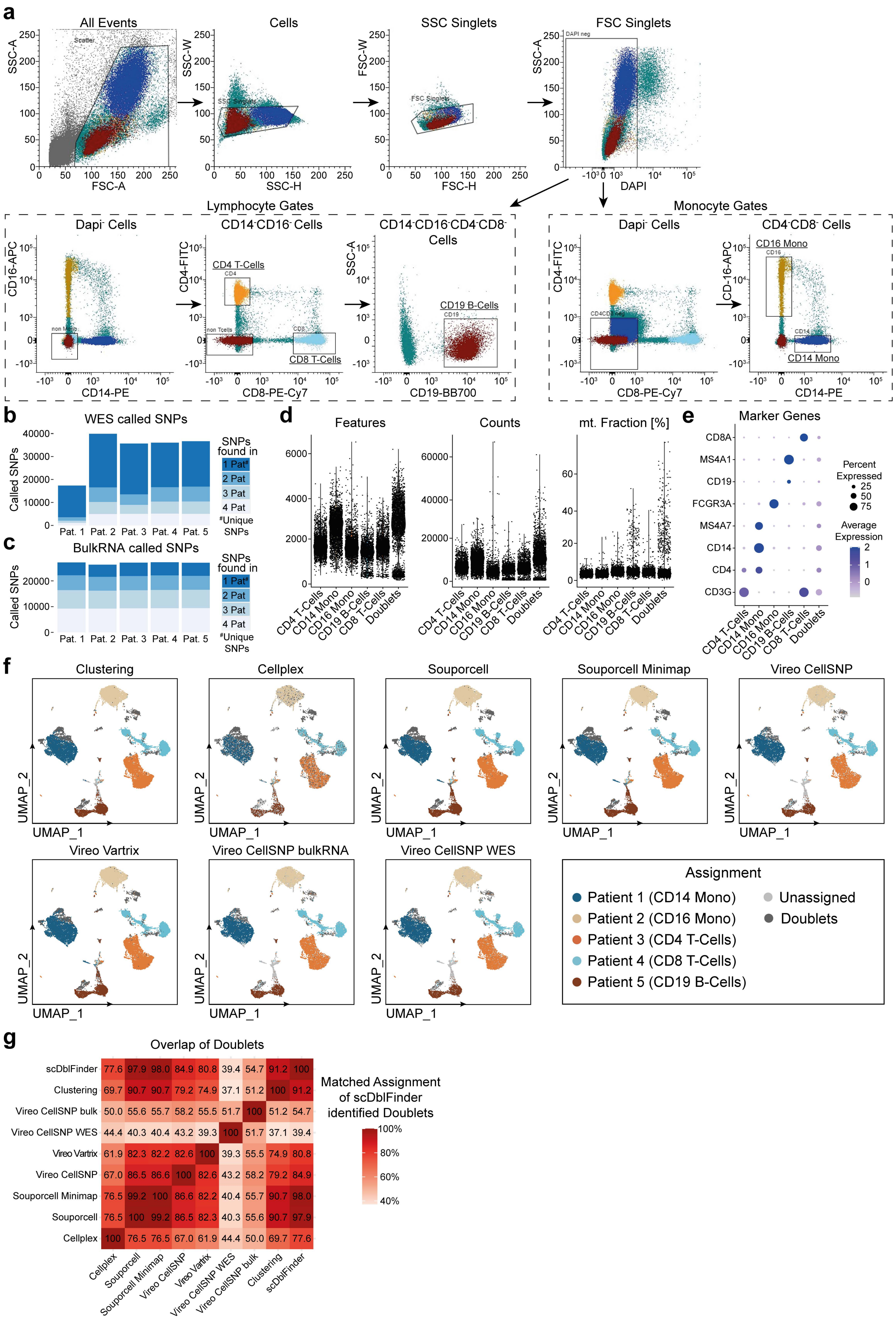
a
All Events
Cells
SSC Singlets
FSC Singlets
SSC-A
SSC-W
FSC-W
FSC-A
SSC-H
FSC-H
DAPI
Lymphocyte Gates
Monocyte Gates
CD14-PE
CD8-PE-Cy7
CD19-BB700
CD16-APC
CD4-FITC
CD4 T-Cells
CD8 T-Cells
CD19 B-Cells
CD16 Mono
CD14 Mono
b
WES called SNPs
Called SNPs
Pat. 1
Pat. 2
Pat. 3
Pat. 4
Pat. 5
SNPs found in
1 Pat#
2 Pat
3 Pat
4 Pat
#Unique SNPs
c
BulkRNA called SNPs
d
Features
Counts
mt. Fraction [%]
CD4 T-Cells
CD14 Mono
CD16 Mono
CD19 B-Cells
CD8 T-Cells
Doublets
e
Marker Genes
CD8A
MS4A1
CD19
FCGR3A
MS4A7
CD14
CD4
CD3G
Percent Expressed
Average Expression
f
Clustering
Cellplex
Souporcell
Souporcell Minimap
Vireo CellSNP
Vireo Vartrix
Vireo CellSNP bulkRNA
Vireo CellSNP WES
UMAP_1
UMAP_2
Assignment
Patient 1 (CD14 Mono)
Patient 2 (CD16 Mono)
Patient 3 (CD4 T-Cells)
Patient 4 (CD8 T-Cells)
Patient 5 (CD19 B-Cells)
Unassigned
Doublets
g
Overlap of Doublets
scDblFinder 77.6 97.9 98.0 84.9 80.8 39.4 54.7 91.2 100
Clustering 69.7 90.7 90.7 79.2 74.9 37.1 51.2 100 91.2
Vireo CellSNP bulk 50.0 55.6 55.7 58.2 55.5 51.7 100 51.2 54.7
Vireo CellSNP WES 44.4 40.3 40.4 43.2 39.3 100 51.7 37.1 39.4
Vireo Vartrix 61.9 82.3 82.2 82.6 100 39.3 55.5 74.9 80.8
Vireo CellSNP 67.0 86.5 86.6 100 82.6 43.2 58.2 79.2 84.9
Souporcell Minimap 76.5 99.2 100 86.6 82.2 40.4 55.7 90.7 98.0
Souporcell 76.5 100 99.2 86.5 82.3 40.3 55.6 90.7 97.9
Cellplex 100 76.5 76.5 67.0 61.9 44.4 50.0 69.7 77.6
Cellplex
Souporcell
Souporcell Minimap
Vireo CellSNP
Vireo Vartrix
Vireo CellSNP WES
Vireo CellSNP bulk
Clustering
scDblFinder
Matched Assignment of scDblFinder identified Doublets
100%
80%
60%
40%

Supplementary Figure 1: a, Representative FACS-Gates and gating strategy for sorted PBMC. Sorted cell population are underlined (CD4 T-Cells, CD8 T-Cells, CD19 B-Cells, CD16 Monocytes, and CD14 Monocytes). Mono: Monocytes. **b**, WES-called SNPs. Pat: Patient. **c**, BulkRNA-called SNPs. **d**, Violin plot of features, counts and mitochondrial gene fraction stratified by clusters. mt: mitochondrial, Mono: Monocytes. **e**, Dotplot of marker genes for each cluster. **f**, UMAP representations of PBMC stratified by clustering or patient assignment from multiplexing methods. **g)** Overlap of doublet assignment for indicated methods of scDblFinder identified doublets (used as a reference). bulk: bulkRNA.

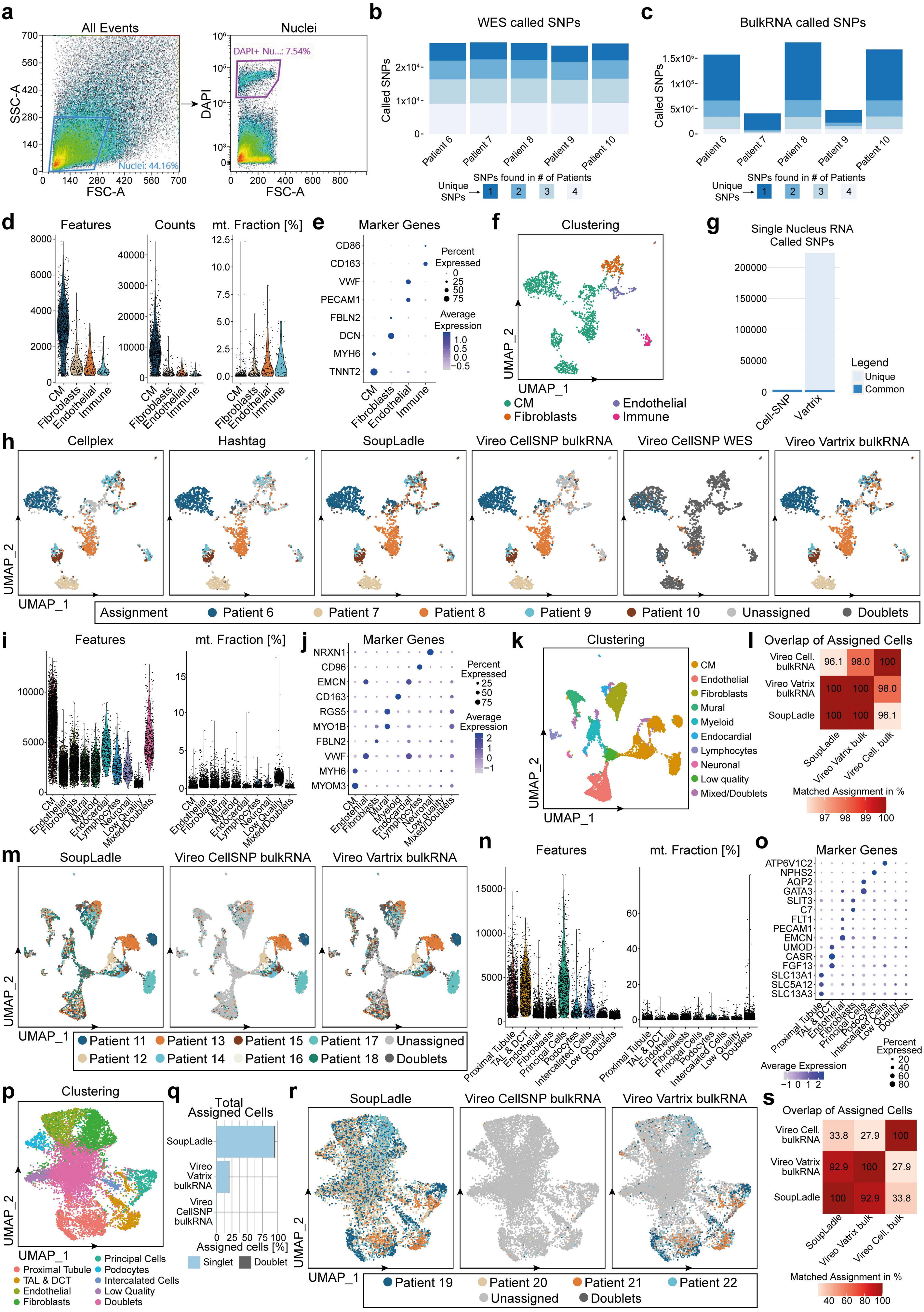
a
All Events
SSC-A
FSC-A
Nuclei: 44.16%
Nuclei
DAPI
DAPI+ Nu...: 7.54%
FSC-A
b
WES called SNPs
Called SNPs
Patient 6
Patient 7
Patient 8
Patient 9
Patient 10
SNPs found in # of Patients
Unique SNPs
1
2
3
4
c
BulkRNA called SNPs
Called SNPs
Patient 6
Patient 7
Patient 8
Patient 9
Patient 10
SNPs found in # of Patients
Unique SNPs
1
2
3
4
d
Features
Counts
mt. Fraction [%]
CM
Fibroblasts
Endothelial
Immune
e
Marker Genes
CD86
CD163
VWF
PECAM1
FBLN2
DCN
MYH6
TNNT2
Percent Expressed
Average Expression
CM
Fibroblasts
Endothelial
Immune
f
Clustering
UMAP_2
UMAP_1
CM
Fibroblasts
Endothelial
Immune
g
Single Nucleus RNA Called SNPs
Legend
Unique
Common
Cell-SNP
Vartrix
h
Cellplex
Hashtag
SoupLadle
Vireo CellSNP bulkRNA
Vireo CellSNP WES
Vireo Vartrix bulkRNA
UMAP_2
UMAP_1
Assignment
Patient 6
Patient 7
Patient 8
Patient 9
Patient 10
Unassigned
Doublets
i
Features
mt. Fraction [%]
CM
Endothelial
Fibroblasts
Mural
Myeloid
Endocardial
Lymphocytes
Neuronal
Low Quality
Mixed/Doublets
j
Marker Genes
NRXN1
CD96
EMCN
CD163
RGS5
MYO1B
FBLN2
VWF
MYH6
MYOM3
Percent Expressed
Average Expression
k
Clustering
UMAP_2
UMAP_1
CM
Endothelial
Fibroblasts
Mural
Myeloid
Endocardial
Lymphocytes
Neuronal
Low quality
Mixed/Doublets
l
Overlap of Assigned Cells
Vireo Cell. bulkRNA
Vireo Vartrix bulkRNA
SoupLadle
96.1
98.0
100
100
100
98.0
100
100
96.1
SoupLadle
Vireo Vatrix bulk
Vireo Cell. bulk
Matched Assignment in %
97
98
99
100
m
SoupLadle
Vireo CellSNP bulkRNA
Vireo Vartrix bulkRNA
UMAP_2
UMAP_1
Patient 11
Patient 12
Patient 13
Patient 14
Patient 15
Patient 16
Patient 17
Patient 18
Unassigned
Doublets
n
Features
mt. Fraction [%]
Proximal Tubule
TAL & DCT
Endothelial
Fibroblasts
Principal Cells
Podocytes
Intercalated Cells
Low Quality
Doublets
o
Marker Genes
ATP6V1C2
NPHS2
AQP2
GATA3
SLIT3
C7
FLT1
PECAM1
EMCN
UMOD
CASR
FGF13
SLC13A1
SLC5A12
SLC13A3
Proximal Tubule
TAL & DCT
Endothelial
Fibroblasts
Principal Cells
Podocytes
Intercalated Cells
Low Quality
Doublets
Average Expression
Percent Expressed
p
Clustering
UMAP_2
UMAP_1
Proximal Tubule
TAL & DCT
Endothelial
Fibroblasts
Principal Cells
Podocytes
Intercalated Cells
Low Quality
Doublets
q
Total Assigned Cells
SoupLadle
Vireo Vatrix bulkRNA
Vireo CellSNP bulkRNA
Assigned cells [%]
Singlet
Doublet
r
SoupLadle
Vireo CellSNP bulkRNA
Vireo Vartrix bulkRNA
UMAP_2
UMAP_1
Patient 19
Patient 20
Patient 21
Patient 22
Unassigned
Doublets
s
Overlap of Assigned Cells
Vireo Cell. bulkRNA
Vireo Vatrix bulkRNA
SoupLadle
33.8
27.9
100
92.9
100
27.9
100
92.9
33.8
SoupLadle
Vireo Vatrix bulk
Vireo Cell. bulk
Matched Assignment in %
40
60
80
100

Supplementary Figure 2: **a**, Representative FACS plots and gating strategy for sorted cardiac nuclei. Sorted nuclei are circled in purple. **b**, WES-called SNPs. **c**, BulkRNA-called SNPs. **d**, Violin plot of features, counts and mitochondrial gene fraction of cardiac nuclei (dataset 1) stratified by clusters. mt: mitochondrial; CM: Cardiomyocytes. **e**, Dotplot of marker genes for each cluster. **f**, UMAP representations of cardiac nuclei stratified by clustering (dataset 1, n=5 pooled). **g**, Quantification of SNPs called from snRNA-seq using CellSNP or VarTrix. **h,** UMAP representations of nuclei stratified by patient assignment from multiplexing methods. **i**, Violin plot of features and mitochondrial gene fraction of cardiac nuclei (dataset 2, n=8 pooled) stratified by clusters. mt: mitochondrial; CM: Cardiomyocytes. **j**, Dotplot of marker genes for each cardiac cluster. **k**, UMAP representations of cardiac nuclei stratified by clustering (dataset 2). **l**, Overlap of cell assignment for multiplexing methods in % of patient assigned cells. bulk: bulkRNA; Cell.: CellSNP. **m,** UMAP representations of cardiac nuclei (dataset 2) stratified by patient assignment from multiplexing methods. **n**, Violin plot of features and mitochondrial gene fraction of kidney nuclei (dataset 3, n=4 samples pooled) stratified by clusters. mt: mitochondrial; TAL & DCT: Thick Ascending Limb and Distal Convoluted Tubule. **o,** Dotplot of marker genes for each kidney cell cluster. **p**, UMAP representations of kidney nuclei stratified by clustering (dataset 3). **q**, Cell assignment (including doublets) in % of all cells. **r,** UMAP representations of nuclei stratified by patient assignment from multiplexing methods. **s**, Overlap of cell assignment for multiplexing methods in % of patient assigned cells. bulk: bulkRNA; Cell.: CellSNP.

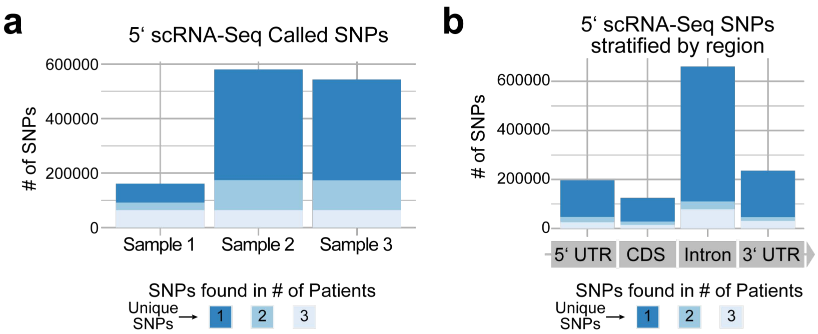

Supplementary Figure 3: a, 5’ scRNA-seq SNPs in three samples from a publicly available scRNA-seq dataset. **b**, Quantification of 5’ scRNA-seq SNPs split by genomic region. CDS: coding sequence; UTR: untranslated region.
