# Supplementary material for: Label-free single-cell RNA multiplexing leveraging genetic variability
Source: Nat Commun. 2024 Dec 5;15:10612. doi: 10.1038/s41467-024-54270-6 (PMC11621319; doi:10.1038/s41467-024-54270-6)
Supplement: Supplementary file 3 — Description of additional supplementary files [file 41467_2024_54270_MOESM3_ESM.pdf]

<u>Description of Additional Supplementary Files</u>

File name: Supplementary Data 1

Description: Estimation of multiplexing costs in comparison to standard 10X scRNA-seq costs

File name: Supplementary Data 2

Description: Exact cell numbers sorted, and cell numbers assigned to patients stratified by multiplexing method
