# Supplementary material for: Label-free single-cell RNA multiplexing leveraging genetic variability
Source: Nat Commun. 2024 Dec 5;15:10612. doi: 10.1038/s41467-024-54270-6 (PMC11621319; doi:10.1038/s41467-024-54270-6)
Supplement: Supplementary file 6 — Reporting Summary [file 41467_2024_54270_MOESM6_ESM.pdf]

nature portfolio

Corresponding author(s): Rafael Kramann, Sikander Hayat

Last updated by author(s): Oct 30, 2024

Reporting Summary

Nature Portfolio wishes to improve the reproducibility of the work that we publish. This form provides structure for consistency and transparency in reporting. For further information on Nature Portfolio policies, see our Editorial Policies and the Editorial Policy Checklist.

Statistics

For all statistical analyses, confirm that the following items are present in the figure legend, table legend, main text, or Methods section.

n/a	Confirmed	
☐	☒	The exact sample size (*n*) for each experimental group/condition, given as a discrete number and unit of measurement
☐	☒	A statement on whether measurements were taken from distinct samples or whether the same sample was measured repeatedly
☐	☒	The statistical test(s) used AND whether they are one- or two-sided<br>*Only common tests should be described solely by name; describe more complex techniques in the Methods section.*
☒	☐	A description of all covariates tested
☒	☐	A description of any assumptions or corrections, such as tests of normality and adjustment for multiple comparisons
☐	☒	A full description of the statistical parameters including central tendency (e.g. means) or other basic estimates (e.g. regression coefficient) AND variation (e.g. standard deviation) or associated estimates of uncertainty (e.g. confidence intervals)
☐	☒	For null hypothesis testing, the test statistic (e.g. *F*, *t*, *r*) with confidence intervals, effect sizes, degrees of freedom and *P* value noted<br>*Give P values as exact values whenever suitable.*
☒	☐	For Bayesian analysis, information on the choice of priors and Markov chain Monte Carlo settings
☒	☐	For hierarchical and complex designs, identification of the appropriate level for tests and full reporting of outcomes
☒	☐	Estimates of effect sizes (e.g. Cohen's *d*, Pearson's *r*), indicating how they were calculated

Our web collection on statistics for biologists contains articles on many of the points above.

Software and code

Policy information about availability of computer code

Data collection	For generation of scRNA and snRNA-seq libraries the following kits were used: PBMC: 10x v3.1. dual index coupled with 3' Cellplex; Heart dataset #1: 10x v3.1. dual index coupled with 3' Cellplex; Heart dataset #2: 10x v3.1 HT dual index; Kidney biopsy dataset: 10x v3.1. dual index.<br>For generation of BulkRNA libraries NEBNext Ultra II Directional Library Prep Kits (NEB, E7760L) were used (with NEBnext rRNA Deleption for PBMC and heart datasets, without rRNA Depletion for kidney dataset).<br>For Whole Exome Sequencing Lotus DNA Library Preparation kit was used (IDT, USA). Samples were sequenced on an Illmuna NovaSeq System.<br>For alignment cellranger (multi) pipeline (version 7.1.0) was used for 10x scRNA/snRNA data. For WES alignment bwa-mem was used, and for bulkRNA STAR alignment was used.
Data analysis	All code has been deposited at github and is available under the following link: https://github.com/ToreBle/SoupLadle [https://doi.org/10.5281/zenodo.13711299]. The packages and software used are as follows:<br>bwa-mem (version 0.7.8), GATK (version 2.3.9), Cellranger (version 7.1.0 with reference genome GRCh38-2020-A), cellSNP-Lite (version 0.3.2), Vireo (version 0.5.7), freebayes (version 1.3.6), VarTrix (version 1.1.22), Souporcell (version 2.4)

For manuscripts utilizing custom algorithms or software that are central to the research but not yet described in published literature, software must be made available to editors and reviewers. We strongly encourage code deposition in a community repository (e.g. GitHub). See the Nature Portfolio guidelines for submitting code & software for further information.

Data

Policy information about availability of data

All manuscripts must include a data availability statement. This statement should provide the following information, where applicable:

- Accession codes, unique identifiers, or web links for publicly available datasets
- A description of any restrictions on data availability
- For clinical datasets or third party data, please ensure that the statement adheres to our policy

Data Availability
Bulk- and scRNA-sequencing data generated in this study have been deposited in the GEO database under accession code GSE247708 [https://www.ncbi.nlm.nih.gov/geo/query/acc.cgi?acc=GSE247708]. Whole exome sequencing data have been deposited in the database of The European Genome-phenome Archive (EGA) with the accession code EGAD50000000928 [https://ega-archive.org/datasets/EGAD50000000928] and are available under restricted access for the protection of patient privacy. Access may be granted to qualified researchers for health/medical/biomedical purposes, who are bound by a Data Use Certification Agreement.

Code Availability:
All original code has been deposited at github and is available under the following link: https://github.com/ToreBle/SoupLadle [https://doi.org/10.5281/zenodo.13711299].

Research involving human participants, their data, or biological material

Policy information about studies with human participants or human data. See also policy information about sex, gender (identity/presentation), and sexual orientation and race, ethnicity and racism.

Reporting on sex and gender	Sex of patients from PBMC cohort: 2 male, 3 female<br>Sex of patients from cardiac tissue cohort 1: 2 male, 3 female<br>Sex of patients from cardiac tissue cohort 2: 5 male, 3 female<br>Sex of patients from kidney biopsy cohort: 3 male, 1 female
Reporting on race, ethnicity, or other socially relevant groupings	Not Applicable.
Population characteristics	PBMC: No relevant co-variate statistics. PBMC samples were collected within the RWTH centralized Biomaterial Bank project without prior criteria for patient inclusion or exclusion. PBMC samples were given to researchers after pseudonymisation and prepped in a blinded fashion.<br>Heart Samples: No relevant co-variate statistics.<br>Kidney Biopsies: No relevant co-variate statistics.
Recruitment	PBMC: No selection criteria were defined for patients.<br>Heart Samples: Snap-Frozen Tissue was collected from patients undergoing left ventricular assist device implantation, total artificial heart or heart transplantation. No exclusion or inclusion criteria were defined for samples.<br>Kidney Biopsies: Kidney biopsies were collected within routine for cause kidney biopsies. No exclusion or inclusion criteria were defined for samples.
Ethics oversight	PBMC Samples: RWTH centralized Biomaterial Bank, Ethics Committee of the Medical Faculty of Medicine of the RWTH Aachen University<br>Heart Samples: Local ethics committee of the Ruhr University Bochum in Bad Oeynhausen (No. 220-640).<br>Kidney Samples: The use of human kidney tissue was approved by the local ethics committee of the Medical Ethics Committee of the Erasmus Medical Center, Rotterdam (MEC-2021-0840).

Note that full information on the approval of the study protocol must also be provided in the manuscript.

Field-specific reporting

Please select the one below that is the best fit for your research. If you are not sure, read the appropriate sections before making your selection.

☒ Life sciences ☐ Behavioural & social sciences ☐ Ecological, evolutionary & environmental sciences

For a reference copy of the document with all sections, see nature.com/documents/nr-reporting-summary-flat.pdf

Life sciences study design

All studies must disclose on these points even when the disclosure is negative.

Sample size	No sample size calculation was performed, sample sizes were determined as n=5-8 based on feasibility of parallel nuclei or PBMC isolation for patients.

Data exclusions	No data was excluded for analysis.
Replication	Multiplexing approach was validated once in PBMC, twice in nuclei isolated from heart tissue and once in nuclei isolated from kidney biopsies.
Randomization	There were no clinical experimental groups (all patients were multiplexed together), therefore not applicable.
Blinding	There were no experimental groups (all patients were multiplexed together), therefore not applicable.

Reporting for specific materials, systems and methods

We require information from authors about some types of materials, experimental systems and methods used in many studies. Here, indicate whether each material, system or method listed is relevant to your study. If you are not sure if a list item applies to your research, read the appropriate section before selecting a response.

Materials & experimental systems

n/a	Involved in the study	
☐	☒	Antibodies
☒	☐	Eukaryotic cell lines
☒	☐	Palaeontology and archaeology
☒	☐	Animals and other organisms
☒	☐	Clinical data
☒	☐	Dual use research of concern
☒	☐	Plants

Methods

n/a	Involved in the study	
☒	☐	ChIP-seq
☐	☒	Flow cytometry
☒	☐	MRI-based neuroimaging

Antibodies

Antibodies used	PE anti-human CD14 Antibody, Dilution 1:100, Clone M5E2, 301850, Biolegend;<br>APC anti-human CD16 Antibody, Dilution 1:100, Clone B73.1, 360705, Biolegend;<br>FITC anti-human CD4 Antibody, Dilution 1:100, Clone RPA-T4, 300506, Biolegend;<br>PE/Cyanine7 anti-human CD8 Antibody, Dilution 1:100, Clone SK1, 344712, Biolegend;<br>BD Horizon™ BB700 Mouse Anti-Human CD19, Dilution 1:100, Clone SJ25C1, 566396, BD Biosciences
Validation	All antibodies have verified reactivity against human epitopes by the original vendors:<br>CD14-AB: https://www.biolegend.com/ja-jp/antibodies-and-more/pe-anti-human-cd14-antibody-796<br>CD16-AB: https://www.biolegend.com/de-de/products/apc-anti-human-cd16-antibody-9053?GroupID=BLG12171<br>CD4-AB: https://www.biolegend.com/ja-jp/soluble-mhc/fitc-anti-human-cd4-antibody-825<br>CD8-AB: https://www.biolegend.com/fr-lu/products/pe-cyanine7-anti-human-cd8-antibody-6390?GroupID=BLG10167<br>CD19-AB: https://www.bdbiosciences.com/en-us/products/reagents/flow-cytometry-reagents/research-reagents/single-color-antibodies-ruo/bb700-mouse-anti-human-cd19.566396

Plants

Seed stocks	Not applicable
Novel plant genotypes	Not applicable
Authentication	Not applicable

Flow Cytometry

Plots

Confirm that:

☒ The axis labels state the marker and fluorochrome used (e.g. CD4-FITC).

☒ The axis scales are clearly visible. Include numbers along axes only for bottom left plot of group (a 'group' is an analysis of identical markers).

☐ All plots are contour plots with outliers or pseudocolor plots.

☒ A numerical value for number of cells or percentage (with statistics) is provided.

Methodology

Sample preparation	PBMC isolation<br>For PBMC isolation blood was collected from five patients (2 male, 3 female) into EDTA-tubes and mixed 1:1 with PBS. EDTA-blood was then carefully layered onto Ficoll-Paque Plus Cytiva (17-440-02, GE Healthcare) (1:1,5) and centrifuged (400 G, 40 min, RT). After centrifugation the PBMC-Layer was aspirated, resuspended in FACS-Buffer (PBS, 2% FCS, 2 mM EDTA, Invitrogen, AM260G) and centrifuged (300 G, 10 min, RT). The supernatant was discarded and cells were resuspended in 300 µl FACS Buffer. At this step, PBMC were counted and ~0.5x106 cells were taken for each, bulkRNA sequencing and WES. The remaining cells were stained with CD14-PE (1:100, Clone M5E2, 301850, Biolegend), CD16-APC (1:100, Clone B73.1, 360705, Biolegend), CD4-FITC (1:100, Clone RPA-T4, 300506, Biolegend), CD8-PE/Cy7 (1:100, Clone SK1, 344712, Biolegend) and CD19-BB700 (1:100, Clone SJ25C1, 566396, BD Biosciences) for 30 min at 4°C, protected from light. Afterwards, cells were washed once with FACS Buffer (400 G, 5 min, 4°C) before staining PBMC with a unique CellPlex-Oligo (3' CellPlex, 1000261, 10X) for each patient (100 µl, 5 min, RT). Subsequently cells were washed twice, before sorting a unique PBMC population for each patient (CD14 Monocytes, CD16 Monocytes, CD4 T-Cells, CD8 T-Cells, CD19 B-Cells) using a BD FACSMelody Cell Sorter. For lymphocytes Dapi, CD16 and CD14 positive cells were excluded to avoid dead cell and monocyte contamination, with subsequent sorting of CD4-CD8+ T-Cells, CD4+CD8- T-Cells or CD4-CD8-CD19+ B-Cells (Supplementary Fig. 1a). For monocytes Dapi, CD4 and CD8 positive cells were excluded to avoid dead cell and lymphocyte contamination, with subsequent sorting of CD14+CD16- or CD16+CD14- monocytes (Supplementary Fig. 1a). For each sample we sorted an equal number of cells (100,000 cells per sample). After sorting, PBMC were pooled and immediately loaded onto a Chromium Next GEM Chip G for snRNA-seq (3' v3.1, 10X) with a cell recovery of ~18000 cells after Cell Ranger alignment.<br><br>Nuclei isolation from snap-frozen tissue<br>Snap frozen tissue was crushed using a mortar and pestle, resuspended in 500 µl nuclei lysis Buffer (EZ lysis Buffer, NUC101, Sigma-Aldrich with 1 Tab/10 ml of cOmplete Protease Inhibitor 11873580001, Roche and 10 µl/ml Recombinant RNase Inhibitor, 2313A, Takara Bio and 10µl/ml Superase In RNase Inhibitor, AM2694, Thermofisher) and homogenized with dounce tissue grinder pestles. The homogenized solution was spun down, supernatant discarded and the pellet resuspended in 4 ml nuclei resuspension buffer (NRB, PBS, 1% BSA, 126615-25ML, Sigma-Aldrich, and 10 µl/ml Protector RNase Inhibitor, 3335399001, Roche, abbreviation: NRB). The suspension was then filtered via a 40 µm cell strainer and centrifuged (500 G, 4° C, 5 min). Supernatant was discarded, and cells resuspended in 200 µl NRBwith 3 µl of a unique TotalSeq anti-Nuclear Pore Complex Antibody Hashtag-Antibody (TotalSeq™ A0451-A0455, Biolegend) for each sample (4°C, 20 min). Subsequently samples were washed with 1 ml NRB, centrifuged (500 G, 4°C, 5 min) and resuspended in 100 µl of a unique CellPlex Oligo (3' CellPlex, 1000261, 10X) for each sample (RT, 5 min). The above steps for Hashtagging and CMO-Labeling were not performed for the larger heart dataset, where eight samples were pooled, and the kidney biopsy dataset, as the additional processing steps led to excessive nuclei loss with insufficient remaining nuclei remaining for adequate chip loading (loading > 10000 cells on the chip). Cells were washed once more with NRB (500 G, 4°C, 5 min) before proceeding to Fluorescent-activated Nuclei-sorting of DAPI positive nuclei with a Sony SH800S. For the first heart dataset (n=5; 2 male, 3 female) complete samples were sorted due to a low amount of recovered nuclei due to the additional required processing steps for Cellplex and Hashtag labeling (exact numbers provided in Supplementary Data 2), while for the second heart dataset an approximately equal number of nuclei per sample was sorted (~50000 nuclei per patient) and subsequently pooled. For kidney biopsies (n=4; 3 male, 1 female), where tissue was scarce, total processed biopsies were pooled prior to sorting to reduce sample loss. 5 minutes prior to sorting, nuclei were stained with DAPI (Sigma-Aldrich). After sorting, nuclei were pooled and immediately loaded onto a Chromium Next GEM Chip G for snRNA-seq (10x, 3' v3.1). The second heart cohort (n=8; 5 male, 3 female) was loaded onto a Chromium Next GEM Chip M for High Throughput snRNA-seq (10x, 3' v3.1).
Instrument	FACS of PBMC: BD FACSMelody Cell Sorter, FACS of Nuclei: Sony SH800S.
Software	Analysis of FACS Data directly with internal software of BD FACSMelody Cell Sorter or Sony SH800S Sorter.
Cell population abundance	PBMC (representative numbers for 1 patient, in % of total events):<br>CD14 Monocytes: 13.01%<br>CD16 Monocytes: 2.97%<br>CD4 T-Cells: 19.52%<br>CD8 T-Cells: 13.53%<br>CD19 B-cells: 5.13%<br><br>Nuclei (representative numbers for 1 patient, in % of parental Gate): FSCvsSSC Nuclei: 44.15%; DAPI+ Nuclei: 7.54%
Gating strategy	PBMC (Extended Data Figure 1a): Gate 1 (FSC-A vs SSC-A): Gating on PBMC. Gate 2 (SSC-H vs SSC-W) and 3 (FSC-H vs FSC-W): Gating on Singlets and exclusion of doublets. Gate 4 (DAPI vs SSC-A): Exclusion of dead cells by gating on DAPI negative cells. From here on, either monocytes or lymphocytes were analyzed. For monocyte analysis and sorting the following strategy was

used: Monocytes, Gate 5 (CD8-PE-Cy7 vs CD4-FITC): After excluding dead cells using DAPI, lymphocytes were excluded by gating on CD4CD8 double negative cells. Monocytes, Gate 6 (CD14-PE vs CD16-APC): Finally, CD16+CD14- (CD16 Monocytes) or CD14+CD16- (CD14 Monocytes) were sorted. For lymphocyte analysis and sorting the following strategy was used: Lymphocytes, Gate 5 (CD14-PE vs CD16-APC): After excluding dead cells using DAPI, monocytes were excluded by gating on CD14CD16 double negative cells. Lymphocytes, Gate 6 (CD8-PE-Cy7 vs CD4-FITC): CD4+ T-Cells were selected by gating on and sorting CD4+CD8- cells. CD8+ T-Cells were selected by gating on and sorting CD4-CD8+ cells. Finally, CD4-CD8- double negative were gated for subsequent selection of CD19 B-cells. Lymphocytes, Gate 7 (CD19-BB700 vs SSC-A): Gating on and sorting of CD19+ B-Cells.

Nuclei (Extended Data Figure 2a): (1) Gating of nuclei based on FSC vs SSC, (2) subsequently gating of DAPI+ nuclei

☒ Tick this box to confirm that a figure exemplifying the gating strategy is provided in the Supplementary Information.
